# Implementation of a horizontal intensive care team can impact efficiency in an emergency observation unit in Brazil

**DOI:** 10.1186/cc14600

**Published:** 2015-03-16

**Authors:** S Ribeiro, C Petrini, L Marino, R Brandao-Neto

**Affiliations:** 1 Hospital das Clinicas School of Medicine, University of São Paulo, Brazil

## Introduction

Emergency department observation units are often used to monitor critical patients in a situation of constant emergency department overcrowding and lack of intensive care beds. However, those units are often understaffed and might not have enough personnel and equipment to provide the same quality of care as a conventional ICU.

## Methods

We performed an observational study in a 17-bed, mixed clinical and surgical emergency department observation. Before the year 2012, the staff were composed of first-year internal medicine and surgical residents. There were no senior physicians specifically assigned to this unit, and residents were supervised by the members of the emergency department team, who changed shifts on a daily basis. In February 2012, two senior physicians (one cardiologist and one intensive care doctor) were specifically assigned to supervise the internal medicine residents and provide horizontal care for medical patients during the day, from Monday to Friday. There was no change in assistance for surgical patients. The schedule for nights and weekends remained unchanged. Mortality and length of stay for medical and surgical patients were measured in 2011, 2012 and 2013.

## Results

In the first year after the implementation of the clinic intensive care team, mortality in internal medicine patients decreased from 47% to 34% in 2012 and 33% in 2013. Although other changes happened in this period (the number of beds decreased from 24 to 17, nurses and physical therapists were hired and trained specifically for this unit), we believe the horizontal staff was critical, because mortality between surgical patients remained almost unchanged in the same period of time (23% in 2011, 22% in 2012 and 23% in 2013), in spite of the structural improvements that equally affected those patients. Length of stay decreased from 6.35 days in 2011 to 3.43 in 2012 and 3.15 in 2013 in medical patients and from 3.9 days on average in 2011 to 3.2 in 2012 and 2.8 in 2013 in surgical patients.

## Conclusion

Emergency department observation units are an alternative to alleviate emergency room overcrowding when there are no intensive care beds available. However, patients end up staying in those units for days and horizontal care by senior doctors may improve outcomes.
